# Applying the Push-Pull Framework to Downsizing in Late Life

**DOI:** 10.1093/geroni/igaa057.1413

**Published:** 2020-12-16

**Authors:** Kyrsten Costlow, Leona Yeager, Shinae Choi, Beverly Roskos, Patricia Parmelee

**Affiliations:** 1 The University of Alabama, Tuscaloosa, Alabama, United States; 2 University of Alabama, Tuscaloosa, Alabama, United States; 3 University of Alabama, Tuscaloosa, Tuscaloosa, Alabama, United States

## Abstract

One of the most frequently cited theoretical models of relocation decision-making is Wiseman’s Behavioral Model of Elderly Migration. Based on this model, the present study used a push-pull framework to describe older adults’ reasons for downsizing to a smaller home. A sample of 68 older adults who had downsized in the past year provided reasons for moving from their previous residence (push factors) and reasons for moving specifically to their new residence (pull factors). Participants rated the importance of each push/pull factor using a (1) not at all important to (4) extremely important scale. On average, participants rated pull factors (M = 3.63, SD = .31) as slightly more important than push factors (M = 3.54, SD = .40) in their decision to move. The most frequently reported push factors were declining health (n = 47, 22%), having too much space or maintenance (n = 40, 19%), and disliking the location of their previous residence (n = 30, 14%). The most commonly reported pull factors were the attractive location of the new residence (n = 60, 28%), social factors (e.g., being closer to family and friends; n = 36, 17%), and services or amenities available in the new residence (n = 32, 15%). Qualitative responses will be presented to illustrate the interaction between push and pull factors in participants’ relocation decision-making. Findings are consistent with those identified in other studies on late-life relocation, establishing Wiseman’s behavioral model as a useful framework for investigating downsizing in late life.

